# The Oral Microbiome in Pediatric IBD: A Source of Pathobionts or Biomarkers?

**DOI:** 10.3389/fped.2020.620254

**Published:** 2021-01-21

**Authors:** Khalid Elmaghrawy, Séamus Hussey, Gary P. Moran

**Affiliations:** ^1^School of Dental Science, Trinity College Dublin and Dublin Dental University Hospital, Dublin, Ireland; ^2^Department of Paediatrics, University of Medicine and Health Sciences, Royal College of Surgeons in Ireland, Dublin, Ireland; ^3^National Children's Research Centre, Dublin, Ireland

**Keywords:** inflammatory bowel disease, Crohn's disease, ulcerative colitis, microbiome, dysbiosis, inflammation, oral cavity

## Abstract

The oral cavity is continuous with the gastrointestinal tract and in children, oral health may be closely linked with the overall health of the GI tract. In the case of pediatric Crohn's disease (CD), oral manifestations are an important clinical indicator of intestinal disease. Recent studies of the microbiome in IBD suggest that translocation of oral microbes to the gut may be a common feature of the microbial dysbiosis which is a signature of both CD and ulcerative colitis (UC). Murine studies suggest that translocation of oral bacteria and yeasts to the lower GI tract may trigger inflammation in susceptible hosts, providing a mechanistic link to the development of IBD. Conversely, some studies have shown that dysbiosis of the oral microbiome may occur, possibly as a result of inflammatory responses and could represent a useful source of biomarkers of GI health. This review summarizes our current knowledge of the oral microbiome in IBD and presents current hypotheses on the potential role of this community in the pathogenesis of these diseases.

## Introduction

In the human body, microbial cells are thought to be at least as numerous as host cells (1–4). The microbiome of the human digestive tract is made up of hundreds of bacterial and fungal species and these microbes harbor 150 times more genes compared to the human genome (5, 6). Microbial population densities in this complex consortium reach their maximum values in the colon, with 10^11^ bacteria per gram of content (4). The gut microbiota play a critical role in human health and have been implicated in nutrient absorption, mucosal barrier fortification, xenobiotic metabolism, angiogenesis and postnatal intestinal maturation (7, 8). Furthermore, the human microbiota stimulates immunity (innate and adapative) and plays an important role in maturation of the immune system (9). Additionally, studies in germ-free mice show that gut microbiota influence body fat deposition, metabolism, and immune function (10, 11). Recent studies suggest that IBD is the result of an altered immune response to the gut microbiota (12). Perturbations in the microbiome may lead to dysbiosis which can be defined as “changes to the structure of a microbial community that are detrimental to its host” (13). This may impair important functions of the microbiome, including its ability to resist pathogenic microorganisms. In the oral cavity, microbiome perturbations caused by increased sugar intake or poor oral hygiene may lead to dental caries and inflammatory gum diseases, respectively (14, 15). Changes to the human gut microbiome have been linked with the prevalence and severity of several diseases, including cancers, IBD and atopic disease (5, 7). The role of the microbiome in the pathogenesis of IBD is still hotly debated, but the disease involves a strong inflammatory response that may be triggered by acquired infection or alternatively by dysbiotic changes to the hosts' own microbiome. In the case of CD, the trigger for this breakdown in homeostasis is unknown, but recent evidence suggests that environmental factors, host genetics and possibly oral-gut transit of microorganisms could all play a role in perturbation of the relationsip between the mucosal immune system and the gut microbiota (16, 17).

## Development of the Oral and Gut Microbiomes in Children

Most of the microbial biodiversity in the human microbiome can be found in the GI tract, and in particular the oral and gut microbiomes (1). However, in terms of composition, these two communities are very distinct. The oral cavity is dominated by facultative, sugar fermenting organisms (e.g., *Streptococcus* and *Actinomyces* spp.) whereas the gut is dominated by a metabolically diverse community of anaerobic bacteria (e.g., *Clostridium* and *Bacteroides* spp.) (1, 18). Although these communities have distinct compositions, the level of species richness is similar in both environments and a single individual may harbor over 100 distinct species at each site (1). Interestingly, the oral microbiome exhibits less interindividual variation compared to the gut microbiome (1). The greater interindividual variation observed in gut microbiomes appears to be related to the greater impact of factors such as diet and antibiotic usage on these communities, whereas as the oral microbiome appears to be more resilient to these challenges (11, 19–21). Both communities are relatively stable over time in healthy individuals (1).

**Figure 1:**
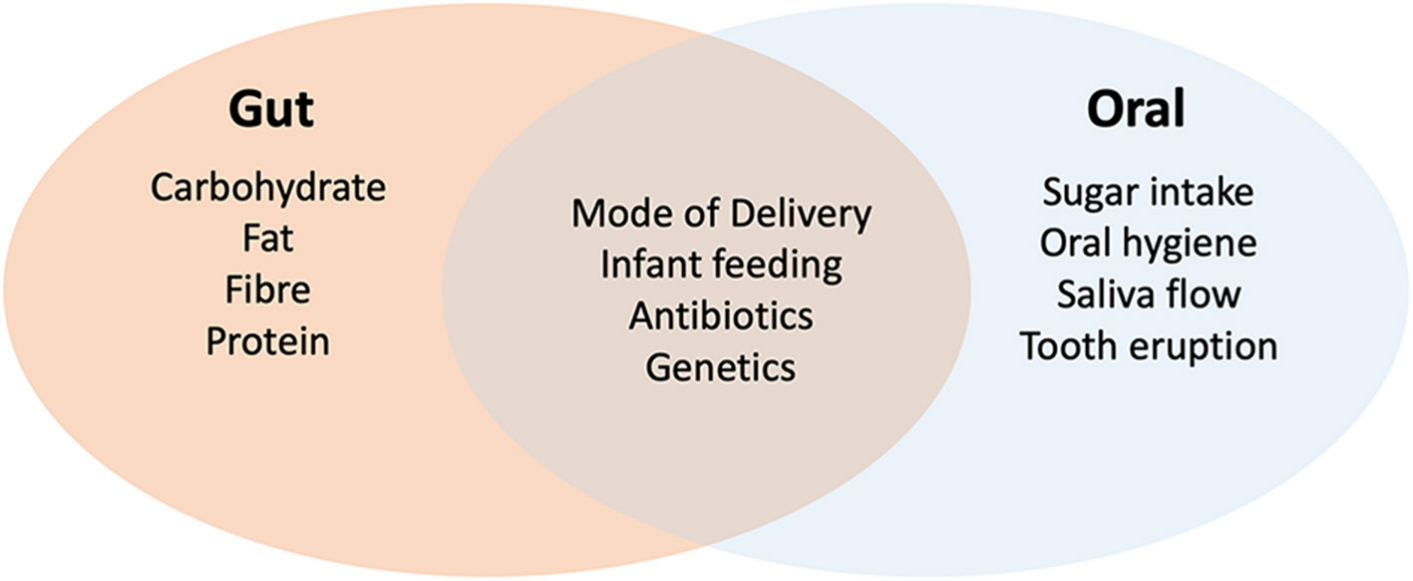
Factors affecting the development of the oral and gut microbiomes in childhood. Evidence shows that both oral and gut microbiomes are influenced by delivery (natural or cesarean), mode of feeding (breast or formula milk) and antibiotics. Genetics may also play a role in colonization. Development of the gut microbiome is heavily influenced by diet whereas sugar intake (especially frequent consumption) has a greater impact on the oral microbiome. In the oral environment, tooth eruption and the level of plaque development (impacted by hygiene and saliva flow) have a major impact on diversity.

The development and maturation of the gut microbiome in infancy is influenced by several factors, including mode of delivery, type of feeding and antibiotic usage (22) (Figure 1). Disturbances to this development have been linked to obesity and atopic disease, suggesting that development of this microbiome has a strong impact on immune maturation and metabolism (23, 24). Some of this regulatory activity has been related to bacterial metabolites including short chain fatty acids (SCFAs) such as acetate, propionate and butyrate. SCFAs are not only an important source of nutrients for enterocytes but also induce differentiation of regulatory T cells (Tregs) which have a central role in the suppression of inflammatory and allergic responses (9, 25, 26). Nutrition also plays an important role in gut microbiome development. In early life, infant breast feeding is associated with reduced biodiversity and high levels of *Bifidobacterium* species capable of metabolizing human milk oligosaccharides (HMOs) (27). The introduction of solid food later results in an increase in biodiversity and the capacity to metabolize more complex carbohydrates. Children exposed to antibiotics display delayed gut microbiome maturation, especially if administered in the first 12 months of life and this can lead to increased levels of *Enterobacteriaceae* and Bacteroidetes (27, 28). The gut microbiome stabilizes at approximately 3 years of age, but diet continues to have a major influence on composition in childhood and later life. Children consuming western diets rich in fats and low in fiber exhibit increased levels of *Bacteroides* species, whereas plant and fiber rich diets are associated with *Prevotella* and *Succinivibrio* (29–31). In general, fat-rich western diets may lead to reduced biodiversity and lower production of SCFAs, which may impact on the development of inflammatory bowel disease (32, 33).

The oral microbiome also develops and matures throughout childhood. The mature oral microbiome is a highly diverse community of up to 700 bacterial species, in addition to the oral fungal mycobiome which is dominated by the yeasts *Candida, Malassezia* and *Saccharomyces* spp. (18, 34–36). The development of this oral microbiome begins shortly after birth and recent data shows that this is also influenced by delivery method and infant feeding (37). Diversity increases with tooth eruption and continues to evolve as children move from decidious to permanent dentition (37, 38). Sugar intake and poor oral hygiene have a major impact on the oral microbiome in children (19, 39). Sugar intake in particular is linked to increased levels of acid tolerant species such as *S. mutans* and *Lactobacillus* spp. (19, 40), the former being directly linked with the pathogeneisis of dental caries. Other dietary factors such as vegetables and refined grain servings have more moderate effects (37, 41). Antibiotic usage in childhood has been shown to affect the oral microbiome, however studies in adults sugest that the oral microbiome recovers more rapidly from antibiotic exposure compared to gut communities (20, 37).

## Oral and Gut Manifestations of IBD

Crohn's disease (CD) and Ulcerative colitis (UC) manifest in different ways. CD is typically characterized by granulomatous inflammation that can affect any part of the gastrointestinal tract (GIT) and involve all mucosal layers, while the inflammation in UC is limited to the colon and only affects the mucosa and superficial submucosa (42–44). The variability in location and the transmural nature of CD leads to a wide variety of presentations and in severe cases, patients may present with systemic symptoms, including fever, anorexia, and weight loss (45, 46). Inflammation in CD most frequently affects the distal ileum and colon but may occur in any part of the gastrointestinal tract including the mouth (47). The term oral Crohn's disease (OCD) is used to describe patients with intestinal CD who exhibit involvement of the oral cavity with a wide variety of disease-specific oral lesions (48). Many oral lesions have been described in CD patients, including swelling of the lips, buccal mucosal swelling or “cobble-stoning,” mucogingivitis, deep linear ulceration, particularly along the buccal gutters, and mucosal tags. Submental lymphadenopathy, perioral erythema with scaling, recurrent buccal abscesses, and angular cheilitis are often observed in patients with CD (43, 48). The oral manifestations of IBD are diverse and based on their relationship with CD activity can be classified as specific (e.g., mucosal swelling) and non-specific oral lesions (e.g., angular cheilitis) (49). Similar oral manifestations have been recorded in different patient cohorts throughout the world (47–52). Although patients with CD can exhibit oral manifestations, pediatric CD has no specific clinical manifestations. Some studies have suggested that oral manifestations are a good cutaneous marker of IBD (50) and are useful diagnostic markers (53).

Orofacial granulomatosis (OFG) is a rare chronic disease that can present with symptoms similar to oral Crohn's disease, including lip swelling and oral inflammation (54). Despite the clinical similarity, OFG patients rarely present with clinical signs of intestinal CD and it is not known if the etiology of OFG is related to that of CD (54). OFG patients have a high incidence of atopy and some studies have suggested the involvement of IgE expressing B-cells in the development of the disease, suggesting that this may involve a hypersensitivity reaction (55, 56).

## The Gut Microbiome in IBD

Significant alterations to the gut and oral microbiomes (discussed below) have been identified in patients with IBD, including both CD and UC patients (12, 17, 51, 57–62). Microbial dysbiosis in the gut of IBD patients has been associated with the generation of a chronic inflammatory response which may be exacerbated by a decrease in community members known to produce immunomudulating SCFAs and increased levels of the *Enterobacteriaceae* (57, 63, 64). Within the last decade, metagenomic profiling microbiome studies have provided detailed information on this dysbiosis and unexpectedly have identified increased abundance of common oral taxa in the gut microbiomes of patients with IBD (e.g., *Veillonella, Haemophilus, Eikenella* spp.) whereas many bacteria with important roles in the generation of SCFAs were reduced (59, 62, 65). An increased H_2_S producers in IBD patients was also noted by Mottawea *et al*. (2016), which was linked to a reduced capacity of mitochondria from IBD patients to detoxify H_2_S, suggesting an additional mechanism for microbiome induced inflammation (60). Studies have shown that this dysbiosis is dynamic and is greatest in patients with severe disease and those with a history of antibiotic usage (59). Medications such as infliximab can also influence the degree of dysbiosis (66–68). The degree of dysbiosis has also been linked to host genetics, with NOD2 risk alleles being shown to be associated with the degree of dysbiosis and the levels of *Enterobacteriaceae* in IBD patients (69, 70).

Changes in the fungal mycobiome have also been reported in IBD patients (71, 72). Fungi are often overlooked because of their low abundance in the lower GIT of humans, however, fungi account for approximately 13% of the gut microbial volume (6, 73). Several investigators have shown an increase in the levels of fungi belonging to the Basidiomycota in IBD patients, including the yeasts of the genus *Malassezia* (74–76). Limon *et al*. (2019) found that the presence of the *Malassezia* spp. in CD showed strong linkage to an allele of the CARD9 receptor (CARD9^*S*12*N*^) associated with CD (76). They went on to demonstrate that *M. restrica* could induce colitis in susceptible mice, providing the first evidence that a member of the normal mycobiome may play a role in the pathogenesis of CD in at least a subset of patients (76).

In summary, the gut microbiome in pediatric IBD is now well-characterized. Several longitudinal, large cohort studies involving treatment naïve patients have clearly shown that changes to the gut microbiome are intrinsically linked to IBD and are responsive to therapy (59, 62, 65). This dysbiosis appears to be associated with reduced biodiversity and reduced levels of normal gut microbes with increases in the levels of *Enterobacteriaceae* and bacterial and yeast species normally associated with the oral cavity (e.g., *Veillonella, Haemophilus, Eikenella* and *Malassezia* spp.).

## Changes in the Oral Microbiota in Patients With Crohn's Disease

A general feature of the studies described above is the identification of high levels of oral taxa in the gut of IBD patients. The oral microbiome is well-characterized in terms of its role in oral diseases (caries, periodontitis), however its members have also been implicated as contribitory factors in several non-oral diseases such as colorectal cancer, diabettes mellitus, cardiovascular disease, bacteremia and preterm birth (77). Relatively few studies have directly investigated the impact of IBD on the oral microbiome in pediatric patients. The first analysis of the oral microbiome in pediatric IBD was carried out using DNA microarrays (51). In this study oral swab samples from the tongue and buccal mucosa of pediatric CD patients on active treatments were examined. Although the species-resolution of DNA microarray analysis is weak compared to more recent sequence based approaches, it was found that the overall microbiome biodiversity of CD patients was significantly reduced compared to healthy children. The tongue samples from pediatric CD patients showed reduced levels of two phyla (Fusobacteria and Firmicutes) and additionally, CD patients with oral manifestations were also shown to have a higher level of anti-Saccharomyces cerevisiae antibody (ASCA) (51).

More recently, 16S sequence based approaches have been used to examine the salivary microbiome in IBD. Said *et al*. (2014) analyzed the salivary microbiome of adult IBD patients (*n* = 35) including both CD and UC patients (61). Significant shifts in the oral microbiome composition of adult CD patients were observed, with higher levels of *Prevotella* and *Veillonella* spp. and reduced *Streptococcus* and *Haemophilus* spp. (61). Furthermore, a study by Xun *et al*. (2018) also examined the salivary microbiome in adult IBD patients (UC = 57 and CD = 13) (78). This analysis revealed increased levels of *Streptococcus* spp. and *Enterobacteriaceae* in UC patients, increased abundance of *Veillonella* spp. in CD patients, accompanied by depletion of *Prevotella, Neisseria* and *Haemophilus* spp. (78). Zhang *et al*. (2020) compared the salivary microbiome of CD patients (*n* = 29) with patients in remission (*n* = 31) and showed that active CD was associated with depletion of *Neisseria, Haemophilus, Fusobacterium* and *Porphyromonas* spp. compared to those patients in remission (79). Taken together, these sequence based studies show that oral dysbiosis occurs in IBD, although the exact nature of the dysbiosis characterized differs between studies. This may be due to the relatively small patient cohorts examined and the use of diverse anti-inflammatory medications in each group. In addition, in the context of pediatric IBD, none have exclusively focused on children and do not include longitudinal elements to determine if treatment is associated with reversal of dysbiosis. Despite these limitations, most studies concur that the genera *Prevotella, Haemophilus* and *Veillonella* are somehow affected in the oral cavity in IBD, which interestingly matches the genera shown to be enriched in the gut microbiota in CD patients (59). The possible link between changes in the oral microbiome and gut microbiota will be discussed further below.

The relationship between OFG oral CD is also under intense investigation and studies of the oral microbiome may indicate if these diseases have similar etiologies. Recently Goel *et al*. (2019) examined the salivary microbiome in 261 subjects comparing the oral microbiome of OFG patients and CD patients (including those with and without OFG) (80). A specific sequence type (oligotype) of *S. salivarius* was found to exhibit increased abundance in individuals with CD or OFG compared to controls and may have promise as an early biomarker of CD (80).

## Oral-Gut Microbial Transit in IBD?

Recent studies of the gut microbiota in pediatric IBD patients have shown an increase in *H. parinfluenza, Veillonellaceae, Neisseriaceae*, and *Fusobacteriaceae* in CD patients (59) and *H. parainfluenza and Veillonella* spp. in UC patients (62). The increased abundance of these taxa in the gut environment may be the result of oral-gut transit. Despite the barrier of the acidic pH of the stomach, oral-gut transit of microorganisms is probably commonplace, as studies of probiotic bacteria show that microrganisms can transit from the oral cavity to the gut and remain viable (81). Auchtung *et al*. (82) also recently showed that yeast in the gut were acquired from the oral cavity and their levels in the gut were associated with dietary consumption (*Saccharomyces*) or poor oral hygiene (*Candida*).

Transit of these microorganisms from the oral cavity may disrupt host-microbiome homeostasis in the gut by displacing commensal SCFA producers. Additionally, some studies indicate that these oral taxa may be directly immunogenic. Previous studies have suggested that two of these bacterial taxa, *H. parinfluenza* and *Veillonella* spp. induce dendritic cell maturation, with the mature dendritic cells producing cytokines which may drive inflammation (83). *Rothia mucilaginosa*, a species associated with ulceration by Gevers *et al*. (59) can produce toxic levels of acetaldehyde in the presence of ethanol, which is a by-product of Enterobacterial fermentations (84). Rengarajan *et al*. (85) also showed significant antibody responses in the colon of IBD patients to bacteria normally found in the oral cavity including *Gemella, Peptostreptococcus*, and *Streptococcus* species.

Experimental evidence for the impact of oral gut transit in IBD patients was reported by Atashari *et al*. (86), who showed that inoculation of the oral microbiome from IBD children to C57BL/6 (B6) germ-free mice resulted in the accumulation of inflammatory IFN-g+ CD4+ TH1 cells. The gut microbiome of these mice was enriched in *Fusobacterium, Veillonella* and *Klebsiella* spp. Subsequent experiments showed that a *Klebsiella pneumonia* strain (Kp-2H7) recovered from the human salivary microbiota was the primary driver of the inflammatory TH1 cell responses, with marked accumulation of TH1 cells in the murine colon. These data clearly show that the oral cavity may be a reservoir for pathobionts, and that trasit from the oral cavity to the gut may play a role in inducing inflammation in IBD patients (86).

## Conclusions and Future Directions

**Figure 2:**
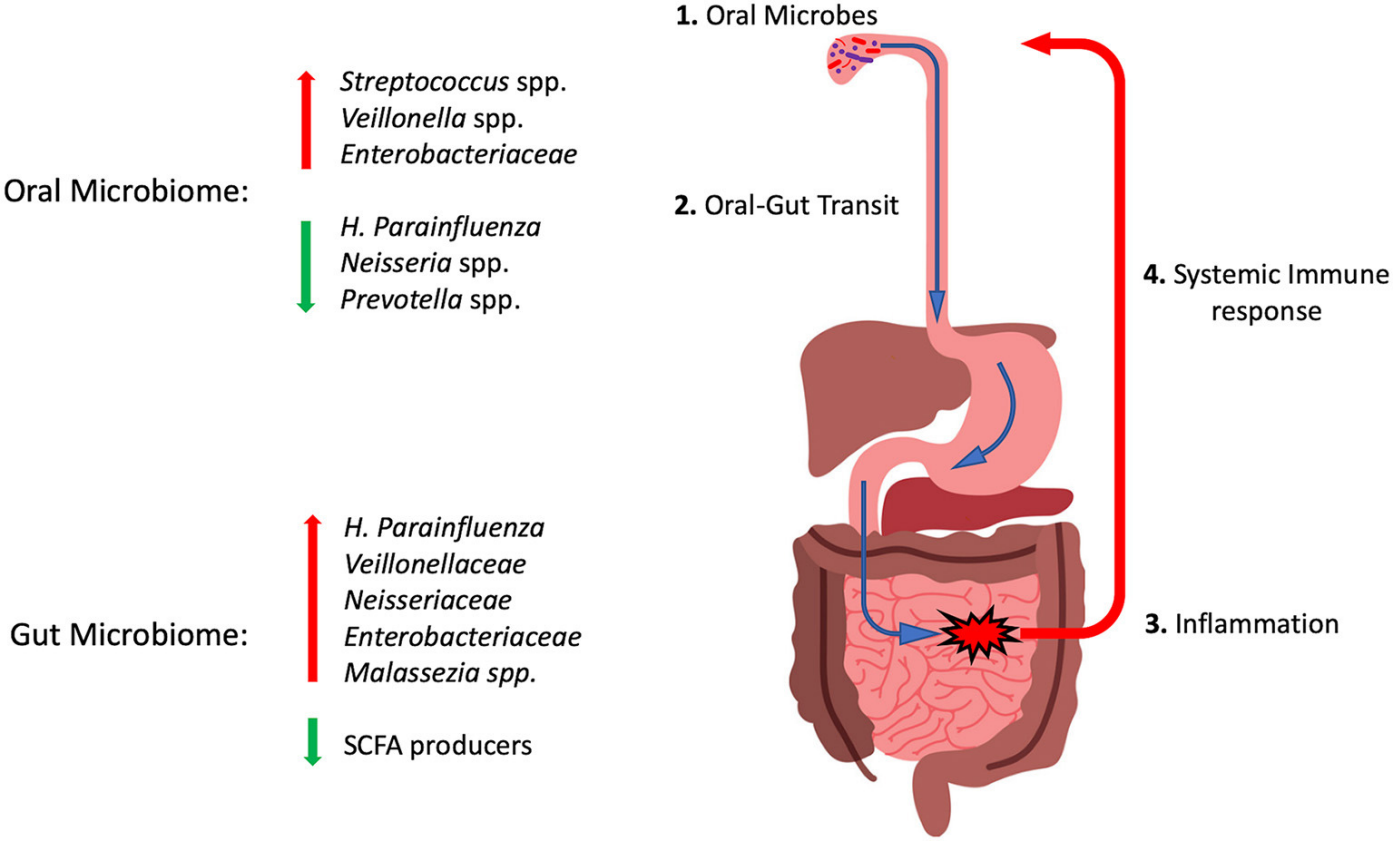
Cartoon diagram summarizing the oral-gut axis hypothesis in pediatric IBD. (1) Microbes in the oral cavity transit through the GI tract (2). In susceptible individuals this may induce inflammatory reactions (3) and displace health promoting SCFA producers. (4) Systemic immune responses to these invading microbes may induce inflammatory reactions that could be involved in generating oral manifestations and altering the abundance of these taxa in the oral microbiome. Red arrows indicate taxa with increased abundance in IBD and green arrows those with reduced abundance. See text for details.

The understandable focus of IBD research to date on the intestinal microbiota has left much to be elucidated regarding the oral microbiome in patients with IBD. Transit of oral microorganisms likely happens in all children, but whether a genetic susceptibility to colonization with pathobionts exists in children with IBD, or whether such taxa are acquired secondarily is unkonwn. It is our hypothesis that oral-gut transit of *Veillonella, Klebsiella* or *Malassezia* spp. could trigger pathobiont-specific systemic responses that could induce inflammatory responses in the oral cavity resulting in oral manifestations of IBD (Figure 2). These systemic reactions may also account for the altered abundance of these species in the oral cavities of IBD patients. The specific nature of this response may make the oral cavity a useful source of biomarkers to diagnose and monitor treatment outcomes in IBD patients. Much of the existing data on the oral microbiome in IBD has come from patients already on various treatments. The effects of chronic treatments or periods of increased disease activity on oral microbial profiles have not yet been studied in sufficient detail. The “holy grail” of biomarker development based on oral sampling remains some time away, and yet would provide a more convenient, accessible and acceptable source for patients and clinicians than current blood or stool specimen-based assays.

## Author Contributions

KE, GPM, and SH all contributed to the literature survey, writing, and editing of the final review.

## Conflict of Interest

The authors declare that the research was conducted in the absence of any commercial or financial relationships that could be construed as a potential conflict of interest.
